# Influencing factors on CPAP adherence and anatomic characteristics of upper airway in OSA subjects

**DOI:** 10.1097/MD.0000000000008818

**Published:** 2017-12-22

**Authors:** Pona Park, Jinil Kim, Yoon Jae Song, Jae Hyun Lim, Sung Woo Cho, Tae-Bin Won, Doo Hee Han, Dong-Young Kim, Chae Seo Rhee, Hyun Jik Kim

**Affiliations:** Department of Otorhinolaryngology, Seoul National University College of Medicine, Seoul, Korea.

**Keywords:** adherence, anatomic characteristics, continuous positive airway pressure, obstructive sleep apnea, upper airway

## Abstract

Although continuous positive airway pressure (CPAP) is the most effective treatment modality, poor adherence still remains a problem for obstructive sleep apnea (OSA) treatment and there is little evidence regarding how this might be improved. This study aims to analyze the anatomic and clinical factors of OSA subjects who failed to comply with CPAP therapy.

The medical records of 47 OSA subjects who received CPAP therapy as a first-line treatment modality were retrospectively reviewed. The medical records were reviewed for demographic and polysomnographic data and anatomic findings of the nasal cavity and oropharynx.

24 patients who adhered to CPAP therapy and 23 patients who were nonadherent were enrolled in the study. There were no statistically significant differences in sleep parameters between CPAP-adherent patients and CPAP nonadherent subjects. Mean body mass index of CPAP nonadherent group was significantly higher than CPAP adherent group. Higher grades of septal deviation and hypertrophic change of the inferior turbinate were observed more in the CPAP nonadherent group. In addition, CPAP nonadherent subjects showed considerably bigger tonsils and higher grade palatal position comparing with the CPAP adherent subjects. Subjective discomfort including inconvenience, mouth dryness, and chest discomfort were the main problems for OSA subjects who did not comply with CPAP therapy.

Excessive upper airway blockage in the nasal cavity and oropharynx was predominant in CPAP nonadherent subjects, which might cause the reported subjective discomfort that reduces CPAP compliance. Therefore, resolution of these issues is needed to enhance CPAP compliance for control of OSA.

## 1 Introduction

Obstructive sleep apnea (OSA) is a common sleep disorder characterized by upper airway collapse, causing reduction or cessation of airflow during sleep. In the Starling resistor model, the upper airway is described as a hollow tube. Within this tube, reduced airflow from the nasal cavity and narrowing of the upper airway increases negative pressure in the pharyngeal airway and predisposes the pharynx to collapse.^[1]^ It has been reported that both upper airway narrowing and increased airway resistance may contribute to the underlying pathogenesis of OSA, leading to symptoms of loud snoring, apnea, and systemic complications if not properly treated.^[2–6]^ Therefore, different therapeutic options, including medical treatment and surgical interventions, have been proposed by researchers to improve upper airway narrowing and to reduce airway resistance in OSA patients.

Treatment modalities for OSA, such as behavioral modification, continuous positive airway pressure (CPAP), surgery, and oral appliances, attempt to widen the upper airway in order to reduce airway collapsibility. CPAP is the standard treatment for patients with moderate-to-severe OSA,^[7]^ and many studies have demonstrated clinical benefits from CPAP therapy including relief from both subjective symptoms and life-threatening conditions.^[8–13]^ Despite of its well-known benefits, technological improvements and efforts to enhance usability, CPAP adherence is generally poor, and its use is often felt to be bothersome. Several studies have investigated CPAP adherence, and it has been shown to vary between 30% and 80%.^[14–16]^ Furthermore, there is little evidence that suggests how CPAP's utility might be improved and approximately 50% of the patients who received CPAP therapy discontinued it within the first year.^[15,16]^ Therefore, identification of clinical factors that are related to CPAP failure or clinical trials to improve CPAP compliance is necessary in OSA patients.

Multiple factors that affect adherence to CPAP therapy include subjective sleep-related symptoms,^[15]^ OSA severity,^[16]^ knowledge of CPAP's effects^[17,18]^ and side effects and discomfort.^[19]^ In addition, anatomic structure might contribute to CPAP adherence and narrow upper airway anatomy is thought to be related to therapeutic PAP pressure and discomfort. Furthermore, it has been reported that CPAP compliance could be improved with optimal management of narrowed anatomic structures through adjunctive upper airway surgery or medication. Sleep apnea surgery for correction of upper airway narrowing may allow for CPAP to be a therapeutic option,^[20,21]^ and some reports state that adherence to CPAP therapy can be improved after nasal and upper airway surgery.^[22,23]^ Therefore, additional studies are needed to evaluate the upper airway anatomy of OSA patients and to measure various parameters in patients who receive CPAP for OSA treatment in order to ultimately improve compliance with CPAP therapy. However, few studies have reported the physical differences between adherent and nonadherent CPAP patients.

Thus, this study aims to evaluate sleep parameters, septal deviation, inferior turbinate hypertrophy, tonsil size, and palatal position in order to assess the anatomic differences of the upper airway in OSA subjects based on adherence to CPAP therapy.

## 2 Materials and methods

### 2.1 Study population

This study retrospectively evaluated the medical records of 126 subjects who were diagnosed with OSA by an attended fulltime polysomnography (PSG) at the sleep center of Seoul National University Hospital from November 2014 to October 2015. The study was approved by the Institutional Review Board of Seoul National University Hospital (SNUH 2016-0325). Patients who were prescribed CPAP therapy as their first OSA treatment modality were considered for inclusion. Medical records were reviewed for demographic data, treatment outcome, sleep parameters of PSG, nasal and oropharyngeal endoscopic findings, and body mass index (BMI). Obesity was defined as a BMI ≥ 25. CPAP compliance was assessed 3 months after initiating CPAP using the machine's built-in compliance meter, and the subjects were divided into CPAP adherent and CPAP nonadherent groups. Demographic data, sleep parameters measured by full-time PSG, and physical examination findings of the nasal cavity and oropharynx were analyzed, and the data were compared between the 2 groups.

### 2.2 Full-night-in-laboratory polysomnography

All the subjects were required to complete a full-night in-laboratory PSG with the supervision of an experienced technician at the sleep center. The device used in this study was the Neuvo PSG device (Compumedics, Victoria, Australia), which contained 13 channels including electroencephalogram, electrocardiogram, oculogram, submental and anterior-tibialis electromyogram, nasal cannula and oral airflow, thoracic and abdominal movement, body position, and oxygen saturation measured by pulse oximetry. Electroencephalography electrodes were placed at C4/A1, C3/A2, O1/A2, and O2/A1. Two electrooculography electrodes were placed at the sides of both eyes to record horizontal and vertical eye movements. Electromyography electrodes were placed at the submentalis muscles and both anterior tibialis muscles. Oxygen saturation was measured using a pulse oximeter applied to the index finger, and nasal pressure cannulas were used to record airflow. Strain gauges recorded chest and abdominal respiratory movements. The respiratory distress index (RDI) identifies the number of apnea or hypopnea events per hour of sleep and respiratory effort related arousal. OSA is diagnosed if 5 or more of these events are identified on PSG. OSA severity was defined as mild for an RDI ≥5/h and <15/h, moderate for an RDI ≥15/h and ≤30/h, and severe for an RDI ≥30/h, as recommended by the 2009 AASM.^[24]^

### 2.3 Continuous positive airway pressure

CPAP therapy was initiated in the outpatient clinic setting as part of routine clinical care. During the first visit, patient education regarding OSA, explanation of treatment risks and benefits, nasal mask fitting and CPAP device instruction were all addressed. After baseline polysomnographic evaluation, a second night of PSG was performed to titrate the CPAP. All subjects received CPAP treatment for at least 3 months and after 3 months of therapy, subject adherence, delivered pressure, air leak levels, and residual events of OSA were assessed. Data on CPAP usage and mean AHI during CPAP treatment were recorded on the CPAP device. No pressure change was made during the study period if patients did not complained regarding CPAP use or if their AHI was less than 5/h. CPAP pressure was adjusted when patients reported problems with CPAP use or their AHI was over 5/h. Patients were considered to be compliant with PAP therapy if they used CPAP for an average of 4 hours each night for at least 70% of nights, and nonadherence was defined as CPAP use for less than <4 hours per night on ≥ 70% of nights or lack of symptomatic improvement (or both) during a period of at least 3 months.^[25]^

### 2.4 Physical examination of the nasal cavity and oropharynx

**Figure 1:**
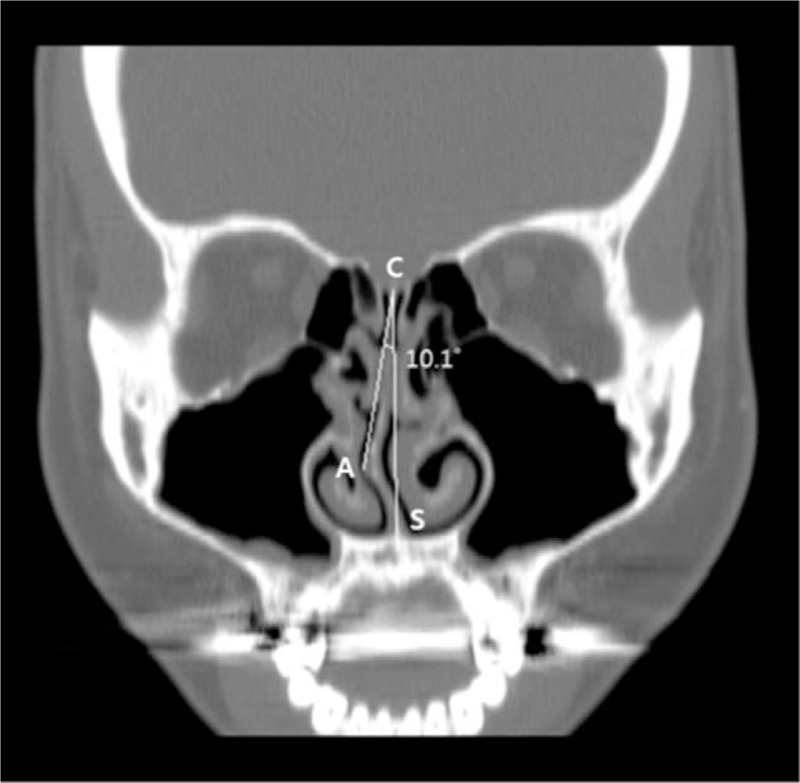
Assessment of septal deviation with computed tomography (CT). The presence of septal deviation was evaluated using coronal CT scans. The superior insertion of the nasal septum at the crista galli (C), its inferior insertion at the level of the anterior nasal spine (S), and the apex of the nasal septal deviation (A) were all marked on the CT scan. The septal deviation angle was defined as the angle between the midline (C–S line) and the line from the crista gali to most markedly deviated point (C–A line). CT = computed tomography.

Nasal endoscopic examination and paranasal sinus computed tomography (PNS-CT) was performed as part of routine clinical care. The presence of septal deviation and inferior turbinate hypertrophy were assessed using coronal CT images. The superior insertion of the nasal septum at the crista galli (C), its inferior insertion at the level of the anterior nasal spine (S), and the apex of the nasal septal deviation (A) were all marked on the CT scan. The septal deviation angle was defined as the angle between the midline (C–S line) and the line from the crista gali to most markedly deviated point (C–A line). The patients were divided into 3 groups according to the measured degree of nasal septum deviation. Deviation was graded as I (<9^°^), II (9^°^–15^°^), and III (>15^°^) according to the previously proposed grading system (Fig. 1).^[26]^ Hypertrophic change of the inferior turbinate was assessed on nasal endoscopic examination and classified by using a 1 to 4 grading scale (I: 0–25% of total airway space; II: 26–50% of total airway space; III: 51–75% of total airway space; IV: 76–100% of total airway space).^[27]^ According to Friedman classification, tonsil hypertrophy was classified in varying degrees from I to IV (I: tonsils inside the tonsillar fossa lateral to posterior pillar; II: tonsils occupy 25% of the oropharynx; III: tonsils occupy 50% of the oropharynx; IV: tonsils occupy 75% or more of the oropharynx, almost meeting in the midline). Palatal length and tongue size were assessed using the modified Mallampati classification into 4 classes: class I (all the oropharynx including tonsils, pillars, soft palate, and the tip of uvula can be easily visible), class II (the tonsils’ upper pole and the uvula are visible), class III (part of the uvula and soft palate are visible), and class IV (just the hard palate and part of soft palate are visible).

### 2.5 Statistical analysis

Statistical differences between sleep parameters and clinical factors in CPAP adherent and nonadherent patients were determined by the Mann–Whitney *U* test or chi-square test, depending on the type of variable. The grade of septal deviation, hypertrophic change of the inferior turbinate, tonsil size, and palatal position were statistically compared using Pearson's chi-square test. All statistical analysis was performed using SPSS (version 18.0; SPSS Inc., Chicago, IL) for Windows software. A *P* value of <.05 was considered statistically significant.

## 3 Results

### 3.1 Clinical characteristics of subjects

One hundred twenty-six subjects were recorded by standard PSG, and 47 of them were prescribed CPAP as their first-line OSA treatment modality. Their average RDI was 42.45/h, which was almost 2 times higher than the average RDI (24.34/h) of patients who were recommended mandibular advancement device (MAD) or surgery as their first-line OSA treatment modality. The average BMI and the lowest SaO_2_of the patients who received CPAP therapy were 29.4 kg/m^2^ and 73.4%, respectively.

**Figure 2:**
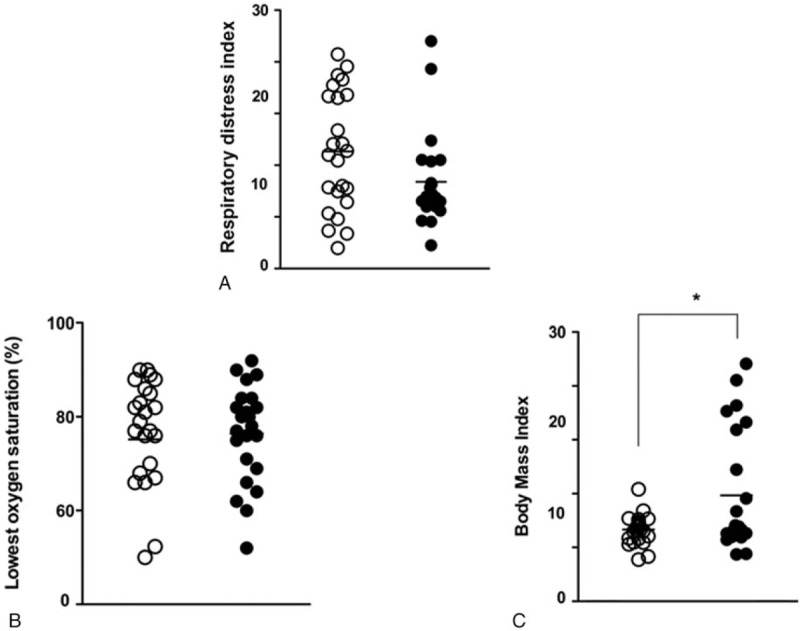
Comparison of RDI (A), the lowest oxygen saturation (B) and BMI (C) between CPAP adherent and nonadherent groups. There was a statistically significant difference in BMI between the 2 groups (∗) (*P* = .021). (^∗^: *P* < .05 when comparing grades between CPAP adherent and nonadherent groups). BMI = body mass index, CPAP = continuous positive airway pressure, RDI = respiratory distress index.

Among those 47 subjects, 24 subjects were included in the CPAP adherent group, and 23 subjects were classified into the CPAP nonadherent group on follow-up. There was no statistically significant difference in sex and age between the 2 groups (Table 1). No statistically significant difference was observed in AHI or the lowest SaO_2_ between the 2 groups (Fig. 2A and B). However, the mean BMI (39.3 kg/m^2^) of patients in the CPAP nonadherent group was significantly higher than that of those in the CPAP adherent group (26.6 kg/m^2^) (*P* < .05, Fig. 2C). The percentage of obese subjects (77%) and the number of patients with a BMI over 30 (N = 8) were greater in the nonadherent group. Through these data, we found that no clinical factors or sleep parameters affect CPAP adherence, but if obese patients received CPAP as their primary OSA treatment, the possibility of CPAP failure was relatively elevated.

**Table 1:**
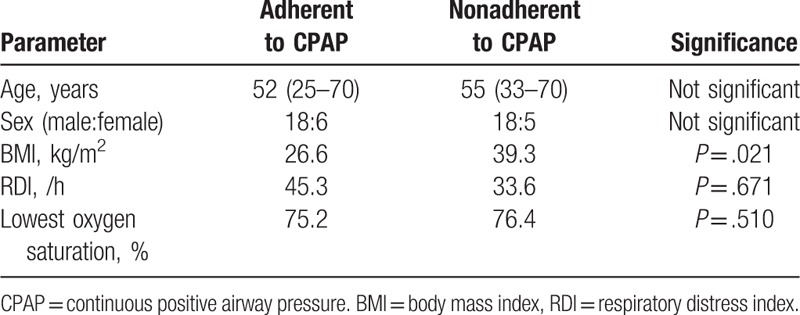
Anthropometric data of patients who were adherent and nonadherent to CPAP.

### 3.2 Endoscopic nasal cavity findings and variables measured by acoustic rhinometry

**Figure 3:**
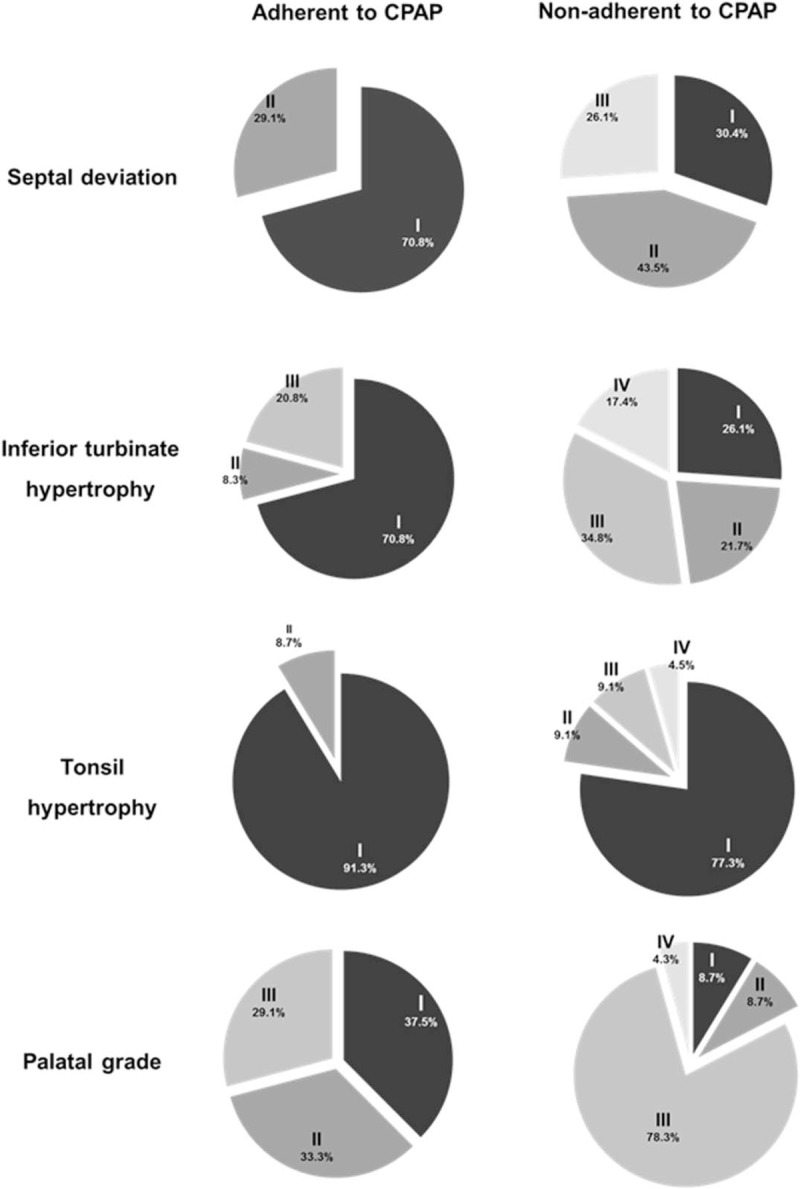
Comparative analysis of anatomic findings of the nasal/oral cavity and the oropharynx between CPAP adherent and nonadherent groups. Comparison of septal deviation (A) and inferior turbinate hypertrophy (B). The number of patients who had septal deviation and inferior turbinate hypertrophy was significantly higher in the CPAP nonadherent group. Evaluation of tonsil size (C) and palatal grade (D) in the oropharynx. Tonsil size in CPAP nonadherent patients was significantly greater than in CPAP adherent patients. The number of patients who had a higher grade of palatal position was significantly higher in the CPAP nonadherent group than in the CPAP adherent group (^∗^: *P* < .05 when comparing grades between CPAP adherent and nonadherent groups). CPAP = continuous positive airway pressure.

As a next step, the anatomic structures of the nasal cavity were evaluated in the CPAP-adherent and nonadherent groups using an intranasal endoscope. The degree of septal deviation and hypertrophic change of the inferior turbinate were graded based on PNS-CT and nasal endoscopic findings and these values were compared in the CPAP adherent and nonadherent groups. We found that 70.8% of CPAP adherent subjects had a minimally deviated nasal septum (grade I), and 29.1% had grade II septal deviation (Fig. 3A). Additionally, 70.8% subjects who were adherent to CPAP showed grade I hypertrophy of the inferior turbinate, while only 8.3% subjects had grade III hypertrophy of the inferior turbinate (Fig. 3B). Interestingly, the classified grades about nasal septum and inferior turbinate were totally distinguished in the subjects who were nonadherent to CPAP. In total, 43.5% of subjects who did not adhere to CPAP showed grade II septal deviation, and 26.1% of subjects showed grade III septal deviation (Fig. 3A); 73.9% of subjects exhibited hypertrophic change of the inferior turbinate beyond grade II, and 17.4% of subjects were diagnosed with grade IV inferior turbinate hypertrophy (Fig. 3B). Differences between the 2 groups in the degree of septal deviation and inferior turbinate hypertrophy were statistically significant (*P* ≤ .0001, *P* ≤ .0001, respectively).

In addition, the minimal cross sectional area (MCA) and the nasal volume were also measured by acoustic rhinometry to determine how dramatic a change took place from the congested to the decongested state. The variables in the subjects who adhered to CPAP were compared to those of CPAP nonadherent subjects at baseline and after application of decongestant. Both baseline MCA and mean nasal volume were not significantly different between the CPAP adherent and nonadherent groups. However, the mean nasal volume in nonadherent subjects was relatively higher after the use of decongestant than the mean volume of CPAP adherent subjects (Table 2).

**Table 2:**
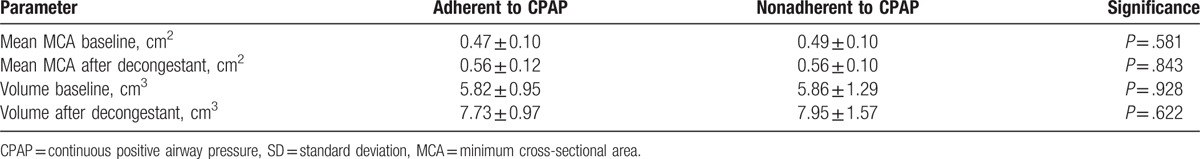
Comparison of variables in acoustic rhinometry between adherent and nonadherent CPAP patients.

Through the results, we found that high-grade septal deviation and more hypertrophic inferior turbinate were observed at a higher rate in subjects who did not adhere to CPAP. Greater volumetric change of the turbinate mucosa was characteristic in CPAP nonadherent subjects, and this change correlated with hypertrophy of the inferior turbinate mucosa.

### 3.3 Comparison of tonsil hypertrophy and palatal position

The subjects’ oropharyngeal structures were also evaluated, and tonsil size and palatal position were graded according to the Friedman and modified Mallampati classification. The results indicated that 91.3% of subjects who were adherent to CPAP showed grade I tonsil hypertrophy and that 8.7% of subjects had more than grade II tonsil hypertrophy. Tonsil size of the subjects who did not adhere to CPAP was significantly greater than that of the subjects in the CPAP adherent group. We found that 22.7% of subjects had grade II, III, and IV tonsil hypertrophy (*P* = .002, Fig. 3C). A palatal position greater than grade II was observed in 91.3% of CPAP nonadherent subjects, which was significantly different when compared to the palatal position of CPAP adherent subjects. Furthermore, the number of subjects who had macroglossia was significantly higher in the CPAP nonadherent group than in the CPAP adherent group (*P* =  < .0001, Fig. 3D). We found that both oropharyngeal structures were distinct between CPAP adherent and nonadherent subjects and that larger tonsils and macroglossia, as determined by high-grade palatal position, were observed in CPAP nonadherent subjects.

### 3.4 Subjective complaints of CPAP nonadherent patients

It has been reported that the local side effects of nasal congestion, dry nose or throat, and discomfort associated with cold air are closely related to CPAP adherence. We considered that these side effects might be due to anatomic abnormalities in OSA patients. As such, we investigated the subjective discomfort of patients in the CPAP nonadherent group through retrospective review of medical records, and we analyzed why they refused CPAP therapy within the first 3 months of treatment.

**Figure 4:**
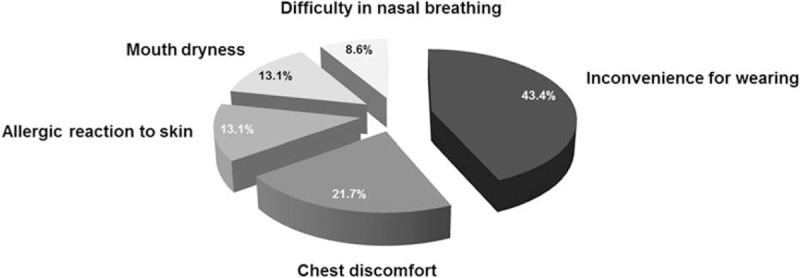
Subjective complaints in CPAP nonadherent patients. There were several subjective complaints from patients in the CPAP nonadherent group. Among them, discomfort while wearing CPAP was the most frequently mentioned complaint. CPAP = continuous positive airway pressure.

Unlike CPAP adherent OSA subjects, the subjects who did not adhere to CPAP complained of more serious discomfort. In total, 43.4% of CPAP nonadherent subjects complained of discomfort while wearing the CPAP, 21.7% refused CPAP therapy due to chest discomfort, and 13.1% complained of dry mouth when they wore the CPAP during sleep. Interestingly, 13.1% of CPAP nonadherent subjects had an allergic reaction to CPAP masks and inevitably stopped wearing CPAP even though their sleep parameters, subjective sleep quality and Epworth sleepiness scale had improved. Another 8.6% patients refused to wear CPAP because it was hard for them to breathe through the nose during sleep (Fig. 4). Among the 23 CPAP nonadherent subjects, 13 patients underwent surgical treatments, 8 patients were treated with MAD, and 2 patients had no desire to continue CPAP therapy and gave up OSA treatment.

## 4 Discussion

**Figure 5:**
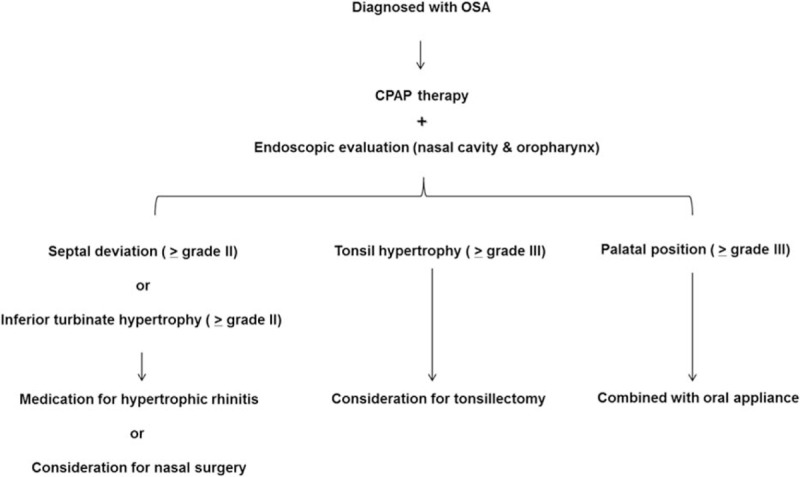
Suggestive schematic picture for evaluation of anatomic factors in OSA subjects who receive CPAP therapy and additional therapeutic approaches according to CPAP adherence. CPAP = continuous positive airway pressure.

The current study demonstrates that anatomic characteristics such as high grade septal deviation, inferior turbinate hypertrophy, larger tonsils and more voluminous tongue are correlated with CPAP compliance. Furthermore, the study shows that assessment of these anatomic factors, including the variables of acoustic rhinometry, can provide distinctive information that predicts whether CPAP will be successful in OSA subjects and proposes that evaluation of anatomic factors in OSA subjects might provide a clinical rationale for additional therapeutic approaches according to CPAP adherence (Fig. 5).

OSA treatment aims to maintain patency of the upper airway during sleep by using devices like CPAP or MAD to enlarge the narrow oropharyngeal airway.^[6–8]^ To the best of our knowledge, CPAP therapy is highly effective for controlling OSA, and it is thought to be the most effective treatment modality for patients with moderate to severe OSA. CPAP treatment, along with lifestyle modifications, can lead to improved cognitive function, cardiovascular stability and improved quality of life.^[8]^ Despite wide documentation of the well-known benefits, poor adherence and prevalent side effects of CPAP therapy have been problematic for OSA subjects, and there is little evidence that suggests how its utility might be improved.^[28,29]^ In addition, poor compliance may lead to suboptimal treatment or nonadherence, and thus, it is important to identify better tolerated treatment options for CPAP.^[30,31]^ In this study, we evaluated anatomic structures and differences in PSG and acoustic rhinometry between OSA subjects who showed the good and poor compliance to CPAP therapy. We also analyzed subjective complaints of CPAP nonadherent subjects as part of an effort to increase CPAP adherence.

Our clinical data showed that the BMI of subjects in the CPAP nonadherent group was significantly higher than the mean BMI of the CPAP adherent group. Obesity may worsen OSA because of fat deposition at specific sites that may affect upper airway obstruction. Weight loss has been suggested as a cornerstone of improvement in OSA treatment outcomes, and many studies sought to assess the effect of CPAP on obese OSA patients. As a result, it has been proposed that obesity-induced hypoventilation could be the reason for resistance to CPAP therapy in morbidly obese patients.^[32–34]^ In the present study, we found that OSA subjects who failed to respond to initial CPAP therapy had higher BMIs, even under optimal treatment conditions. Therefore, we could infer that the more obese OSA subjects might benefit less from CPAP therapy than less obese subjects in the CPAP adherent group. This can in turn lead to poor CPAP adherence, and thus, weight reduction could help raise CPAP adherence in obese OSA patients.

Many anatomical factors may cause upper airway collapse and induce loud snoring or apneic events resulting in OSA. In particular, the disturbance of natural nasal airflow can enhance negative pressure of intraluminal area within the upper airway and trigger airway collapse at the level of the oropharynx or hypopharynx.^[35,36]^ Abnormal anatomic structures of the nose, such as septal deviation, rhinosinusitis with polyps, benign tumors, chronic hypertrophic change of turbinate, and even malignant cancers, may cause or aggravate symptoms of OSA due to serious reduction of nasal airflow and elevated nasal airway resistance.^[37]^ Previous reports have shown that nasal obstruction in normal individuals may lead to an increase in airway resistance at upper airway, which causes the symptoms related with sleep disordered breathings like snoring, apnea, and hypopnea.^[38–40]^ Therefore, we estimated that nasal surgeries for correction of pathologic anatomic factors into the nasal cavity would be critical to reduce nasal airway resistance in OSA subjects.^[41]^ In the present study, we found that a higher number of CPAP nonadherent subjects had high-grade septal deviation and a more hypertrophic inferior turbinate in comparison with CPAP adherent subjects. In particular, 70.8% of CPAP adherent subjects had grade I septal deviation and minimal inferior turbinate hypertrophy when they began CPAP therapy. However, the percentage of those with grade II septal deviation and with higher grade of inferior turbinate hypertrophy was significantly higher in CPAP nonadherent subjects. In addition, acoustic rhinometry showed that the volumetric change of turbinate mucosa after decongestant therapy greater in CPAP nonadherent subjects. This data suggests that a hypertrophic inferior turbinate might and more severe septal deviation be more significant in the nasal cavity of CPAP nonadherent subjects. We presume that the abovementioned nasal pathologies restrict natural airflow through the nasal cavity and that subjects with these anatomic structures need excessive pressure, causing CPAP therapy to be intolerable.

Anatomic factors in the pharynx like enlarged tonsils, macroglossia, redundant pharynx muscles, or narrowing at the glottis can also increase oropharyngeal and hypopharyngeal airway resistance. In the present study, we observed that CPAP compliance was markedly reduced in subjects who had larger tonsils and a higher grade palatal position due to macroglossia. We conclude that the OSA subjects with larger tonsils and larger tongues could have greater airway resistance, which contributes to upper airway collapse during sleep. These anatomic differences necessitate higher CPAP pressure, which reduces CPAP compliance and there have been reports that nasal and upper airway reconstructive surgeries could decrease mean CPAP pressure or improve CPAP adherence.^[22,23,28]^ We believe that improving nasal patency and decreasing excessive upper airway resistance after evaluation of upper airway anatomy may enhance CPAP compliance.

Most OSA subjects in the CPAP nonadherent group complained of subjective discomfort such as physical discomfort when wearing the CPAP, chest discomfort, dry mouth, and nasal obstruction. Some patients suffered from an allergic reaction to the mask. On the other hand, there was no statistically significant difference in AHI and the lowest SaO_2_ between the CPAP adherent and nonadherent groups. It was stated that adherence to CPAP therapy may be more significantly influenced by subjective factors than by objective data obtained using PSG.^[42]^ Therefore, relieving subjective discomfort could be a key factor in improving adherence to CPAP therapy. Recently, many studies have looked at a variety of interventions to increase CPAP compliance. Cvengros et al^[43]^ validated an adaptive intervention strategy in which some subjects received cognitive-behavioral intervention after initial education and it was also reported that CPAP adherence could be improved with ongoing provider follow-up.^[44]^ Although the present study has a limitation that the sample size of the study was small, despite screening of a large number of subjects (N = 126), our data suggests that accurate evaluation of upper airway narrowing in the nasal cavity and oropharynx might be critical for increasing the success rate of CPAP therapy and that it may also reveal the predictive factors for CPAP adherence before starting therapy.

In summary, the current study showed that OSA subjects with serious obesity, higher grade septal deviation, more hypertrophic inferior turbinate, severe tonsil hypertrophy and a higher level of palatal grade did not adhere to CPAP therapy, as these factors induce excessive upper airway narrowing irrespective of sleep parameters. Thus, thorough physical examination at the level of nasal cavity or oropharynx before prescribing CPAP therapy and sufficient explanation of the possible subjective discomfort associated with CPAP treatment is necessary. We also propose that additional therapeutic trials for improving upper airway narrowing before initiation of CPAP therapy could enhance CPAP adherence.
